# Role of ferroptosis-related genes in periodontitis based on integrated bioinformatics analysis

**DOI:** 10.1371/journal.pone.0271202

**Published:** 2022-07-28

**Authors:** Shujian Zhang, Han Jin, Junlong Da, Kai Zhang, Lixue Liu, Yuyao Guo, Wenxuan Zhang, Yawei Geng, Xinpeng Liu, Jiahui Zhang, Lili Jiang, Haoze Yuan, Jianqun Wang, Yuanbo Zhan, Ying Li, Bin Zhang

**Affiliations:** 1 Heilongjiang Provincial Key Laboratory of Hard Tissue Development and Regeneration, The Second Affiliated Hospital of Harbin Medical University, Harbin, China; 2 Heilongjiang Academy of Medical Sciences, Harbin, China; 3 Department of Periodontology and Oral Mucosa, The Second Affiliated Hospital of Harbin Medical University, Harbin, China; Nathan S Kline Institute, UNITED STATES

## Abstract

**Background:**

Cell survival or death is one of the key scientific issues of inflammatory response. To regulate cell death during the occurrence and development of periodontitis, various forms of programmed cell death, such as pyroptosis, ferroptosis, necroptosis, and apoptosis, have been proposed. It has been found that ferroptosis characterized by iron-dependent lipid peroxidation is involved in cancer, degenerative brain diseases and inflammatory diseases. Furthermore, *NCOA4* is considered one of ferroptosis-related genes (FRGs) contributing to butyrate-induced cell death in the periodontitis. This research aims to analyze the expression of FRGs in periodontitis tissues and to explore the relationship between ferroptosis and periodontitis.

**Method:**

Genes associated with periodontitis were retrieved from two Gene Expression Omnibus datasets. Then, we normalized microarray data and removed the batch effect using the R software. We used R to convert the mRNA expression data and collected the expression of FRGs. Gene Ontology (GO), Kyoto Encyclopedia of Genes and Genomes (KEGG), transcription factor (TF) and protein-protein interaction (PPI) network analyses were used. In addition, we constructed a receiver operating characteristic curve and obtained relative mRNA expression verified by quantitative reverse-transcription polymerase chain reaction (PCR).

**Results:**

Eight and 10 FRGs related to periodontitis were upregulated and downregulated, respectively. GO analysis showed that FRGs were enriched in the regulation of glutathione biosynthetic, glutamate homeostasis, and endoplasmic reticulum-nucleus signaling pathway. The top TFs included *CEBPB*, *JUND*, *ATF2*. Based on the PPI network analysis, FRGs were mainly linked to the negative regulation of IRE1-mediated unfolded protein response, regulation of type IIa hypersensitivity, and regulation of apoptotic cell clearance. The expression levels of *NCOA4*, *SLC1A5* and *HSPB1* using PCR were significantly different between normal gingival samples and periodontitis samples. Furthermore, the diagnostic value of FRGs for periodontitis were “Good”.

**Conclusions:**

We found significant associations between FRGs and periodontitis. The present study not only provides a new possible pathomechanism for the occurrence of periodontitis but also offers a new direction for the diagnosis and treatment of periodontitis.

## Introduction

Periodontitis is characterized by pathological loss of the periodontal ligament and alveolar bone. It is affected by multiple actions of herpes viruses, bacterial pathogens and immune responses [1]. Tissue destruction in periodontitis is considered to be due to an excessive inflammatory response to bacterial plaque. When protecting the host from microbial invasion, polymorphonuclear neutrophils can cause an upregulated response of reactive oxygen species (ROS) [2–5]. ROS plays a key role in periodontitis due to excessive lipid peroxidation and tissue damage [6,7].

Ferroptosis is a form of cell death that depends on iron regulation; it is caused by the accumulation of active oxygen in lipids and loss of activity of the lipid repair enzyme *GPX4* [8]. Furthermore, ferroptosis has different molecular characteristics from other forms of cell death [9]. There are two main pathways of ferroptosis, the transporter-dependent pathway and the enzyme-regulated pathway [10]. Data from several studies suggest that the biochemical hallmarks of ferroptosis include accumulation of cellular iron, induction of lipid peroxidation, and loss of antioxidant defense [11]. Ferroptosis has been associated with various pathological conditions such as tumor [12], degenerative diseases [13], ischemia–reperfusion injury [14], and inflammatory diseases [15]. Previous studies have determined that 24 genes play a key role in regulating ferroptosis [16]. These genes are defined as ferroptosis-related genes (FRGs) [17]. In general, the main ferroptosis-inducing event is lipid peroxidation. Interestingly, the characteristics of periodontitis include increased metabolites of lipid peroxidation, alveolar bone resorption, and increased inflammatory factors [18]. However, whether the periodontal pathogens can induce ferroptosis has rarely been reported.

Based on the results of previous studies [19,20], we designed the present study to analyze the effects of ferroptosis on periodontitis using bioinformatics methods and found that there is a clear association between ferroptosis and periodontitis.

## Methods

### Microarray data

Two microarray datasets of Periodontitis—GSE16134 and GSE10334—were downloaded from the GEO database (http://www.ncbi.nlm.nih.gov/geo/), using the following keywords: “periodontitis,” and “Homo sapiens.” GSE16134 and GSE10334 were submitted by Demmer RT, Pavlidis P, Papapanou PN. Since these were public datasets, it is impossible to obtain comprehensive personal information, which appears to be a potential limitation. The data obtained from GEO is shown in Table 1.

**Table 1:** The data obtained from GEO.

GEO gene set ID	GSE16134	GSE10334
Platform	GPL570: [HG-U133_Plus_2] Affymetrix Human Genome U133 Plus 2.0 Array	
Number of Affected sites vs. Unaffected sites	241 vs. 69	183 vs. 64
Clinical Data		
Affected sites	Probing Depth > 4 mm, Clinical Attachment Loss ≥ 3 mm, with Bleeding on Probing	Probing Depth > 4 mm, Clinical Attachment Loss ≥ 3 mm, with Bleeding on Probing
Unaffected sites	Probing Depth ≤ 4 mm, Clinical Attachment Loss ≤ 2 mm, no Bleeding on Probing	Probing Depth ≤ 4 mm, Clinical Attachment Loss ≤ 2 mm, no Bleeding on Probing
Smoking	Not	Not

### Normalized data and batch effect removed

The normalization of data was realized by log2 transformation. PreprocessCore package (R version: 3.4.1) was used to normalize the microarray data. Then we converted the probes into gene symbols according to the GEO annotation information. The probes containing multiple genes were integrated by averaging. Variance stabilized counts was used to control initial quality and individual horse effect removed by the Remove Batch Effect function of limma R package.

### Enrichment analyses

The ferroptosis-related genes were uploaded to the ClueGo (version: 2.5.7). P <0.05 indicates a significant difference. In order to confirm the underlying function, the differentially expressed genes (DEGs) were analyzed through GO functional enrichment. GO is a representative and standardized work platform for terminology description or word meaning interpretation of gene and gene product characteristics. The role of FRGs in healthy tissues was observed by Metascape (https://metascape.org). We used the Cluster Profiler package (R version: 4.0.3) to analyze the GO function of DEGs. KEGG is known for "understanding the advanced functions of biological systems and a library of utility programs". KOBAS 3.0 (http://kobas.cbi.pku.edu.cn/kobas3) was used to enrich the KEGG pathway [21]. The box plot was implemented by the ggplot2 package; PCA graphs were drawn by the ggord package; the heat map was displayed by the pheatmap package.

### ChIP-X enrichment analysis 3 (ChEA3)

ChEA3 (https://amp.pharm.mssm.edu/ChEA3) is a tool to find TF targets and sort them [22]. We can use it to obtain related biological functions of FRGs.

### PPI network construction and module analysis

We used the STRING (version: 11.0) (http://string-db.org) to construct a PPI network of the FRGs and the DEGs. When the composite score is> 0.4, the interaction is significant. More in-depth analysis of the interactive network can be realised by the Cytoscape (version: 3.8.2).

### The diagnostic value of FRGs for periodontitis

We established a ROC curve by MedCalc (version: 20.0.3). And area under the curve (AUC) means the diagnostic value of these hub genes for periodontitis. The diagnostic efficacy represented by AUC is divided into three levels: average (0.5–0.7), good (0.7–0.9), and excellent (0.9–1.0).

### Collection of tissue specimens

6 diseased and 6 healthy gingival tissues specimens were obtained from patients in The Second Affiliated Hospital of Harbin Medical University. (Harbin, People’s Republic of China). All patients understand the nature of this study. The Ethics Committee of The Second Affiliated Hospital of Harbin Medical University reviewed and approved this study. We obtained informed written consent.

### Real‐time quantitative PCR (qRT-PCR)

RNA was extracted from gingival samples using RNAiso Plus (TaKaRa, China). Then we used cDNA synthesis kit (TIANGEN, China) to reverse transcription. The primers for qRT-PCR were supply by GENERAL BIOL (China). The comparative 2 −ΔΔCt method was used for relative quantification. The primer sequences were described by Table 2. The analysis were calculated using GraphPad (version: 8.0.1). Statistical significance between two groups was evaluated by t test (p < 0.05).

**Table 2:** PCR primer sequences.

Gene	Forward Primer (5’ to 3’)	Reverse Primer (5’ to 3’)
*NCOA4*	GAGGTGTAGTGATGCACGGAG	GACGGCTTATGCAACTGTGAA
*HSPB1*	ACGGTCAAGACCAAGGATGG	AGCGTGTATTTCCGCGTGA
*SLC1A5*	GAGCTGCTTATCCGCTTCTTC	GGGGCGTACCACATGATCC

## Result

### Identification of up/downregulated DEGs and FRGs

**Fig 1:**
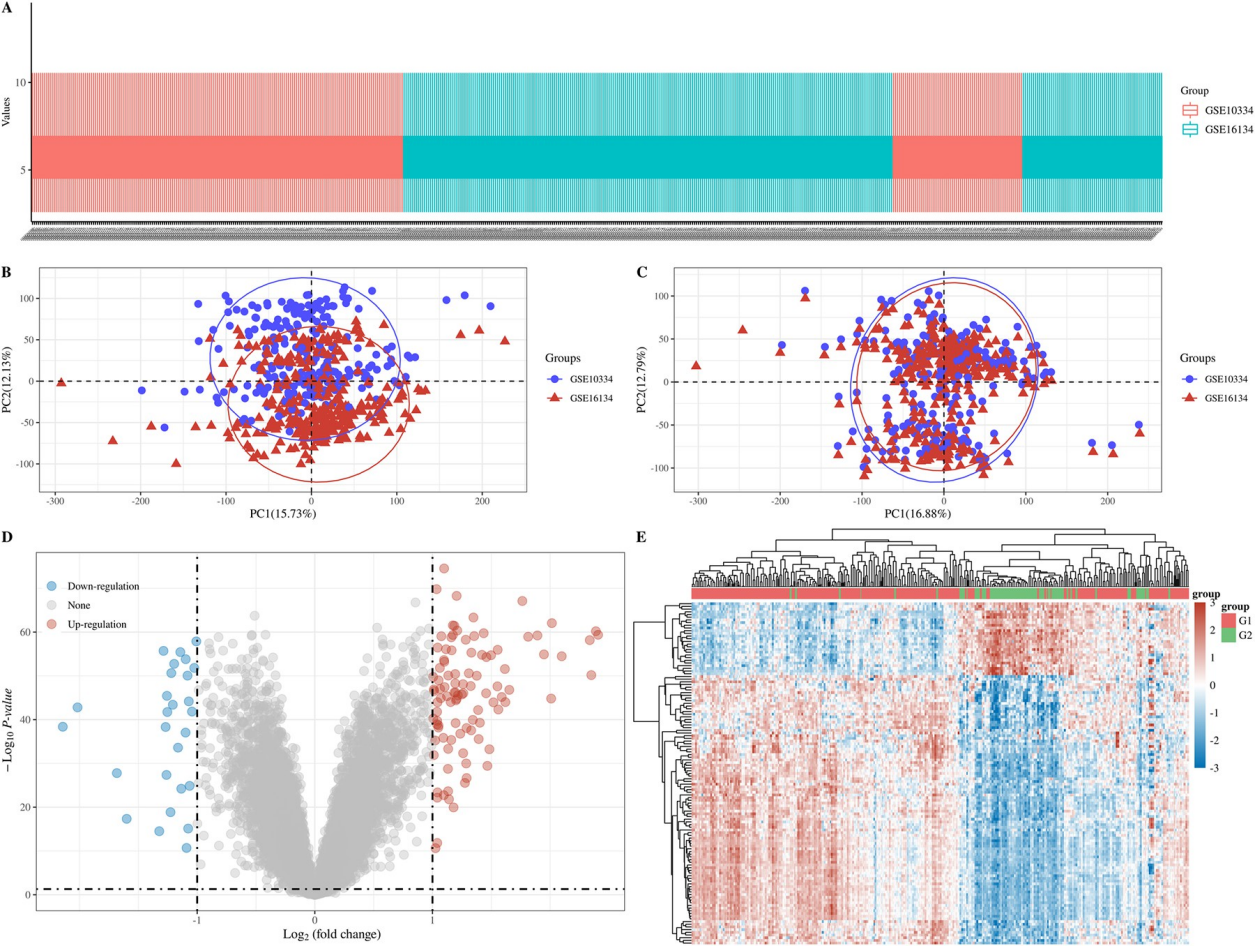
A: Box plot, GSE10334 is shown in red and GSE16134 is shown in blue; B: PCA results before batch removal; C: PCA results after batch removal; D: Volcano plots, and |log_2_(FC)|>1, the over-expressed mRNAs are shown in red and the down-expressed mRNAs are shown in blue, the ordinate represents -log_10_ *P-value*; E: Hierarchical clustering analysis of mRNAs, different colors represent different expression trends, G1: Affected; G2: Unaffected.

**Fig 2:**
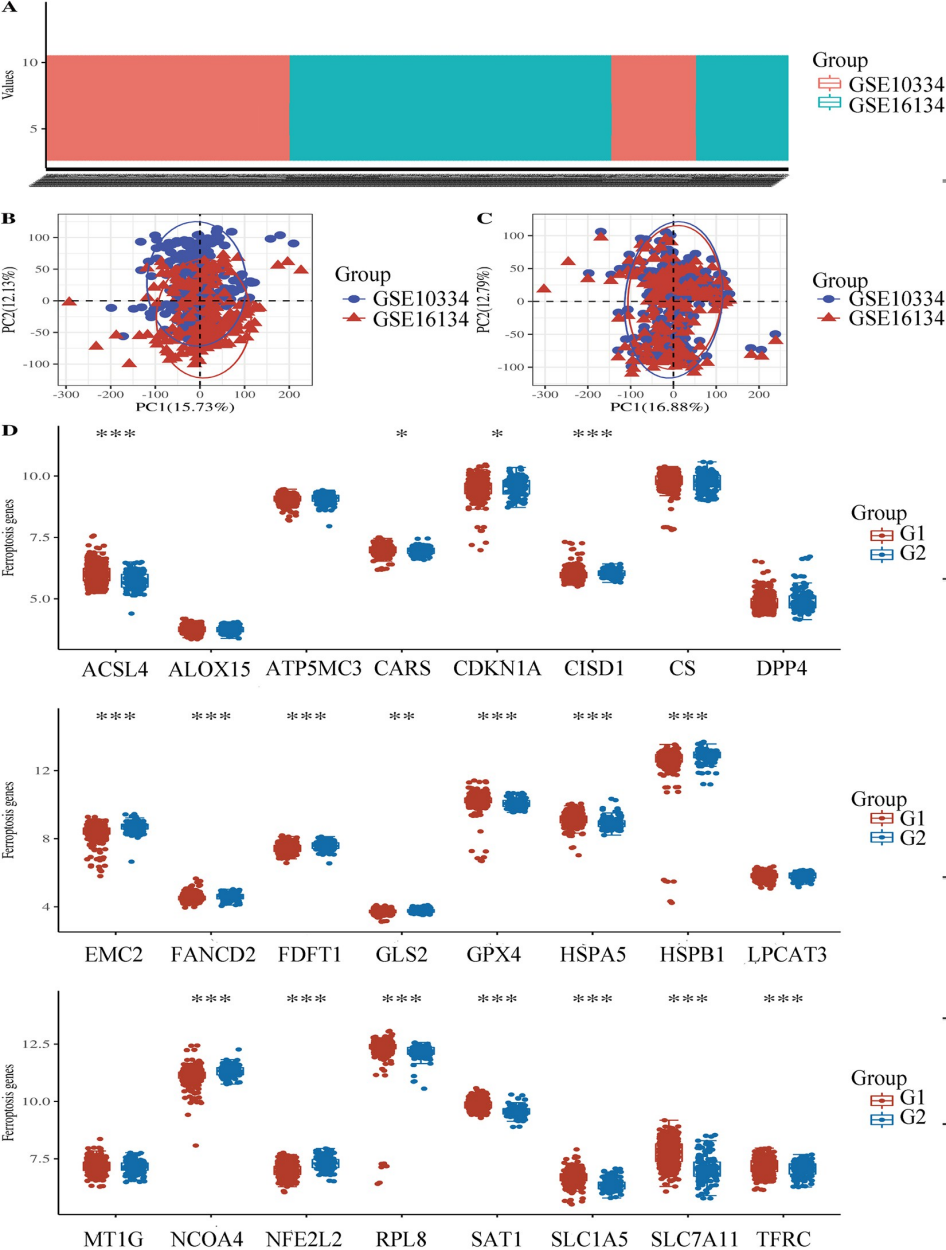
A: Box plot, GSE10334 is shown in red and GSE16134 is shown in blue; B: PCA results before batch removal; C: PCA results after batch removal; D: The expression distribution of Ferroptosis-related mRNA in case and control groups, where the horizontal axis indicates the name of mRNA, the vertical axis indicates the expression of mRNA, and the upper left corner represents the significance p-value test method. Asterisks represent levels of significance *p-value < 0.05, **p-value < 0.01, ***p-value < 0.001.

**Fig 3:**
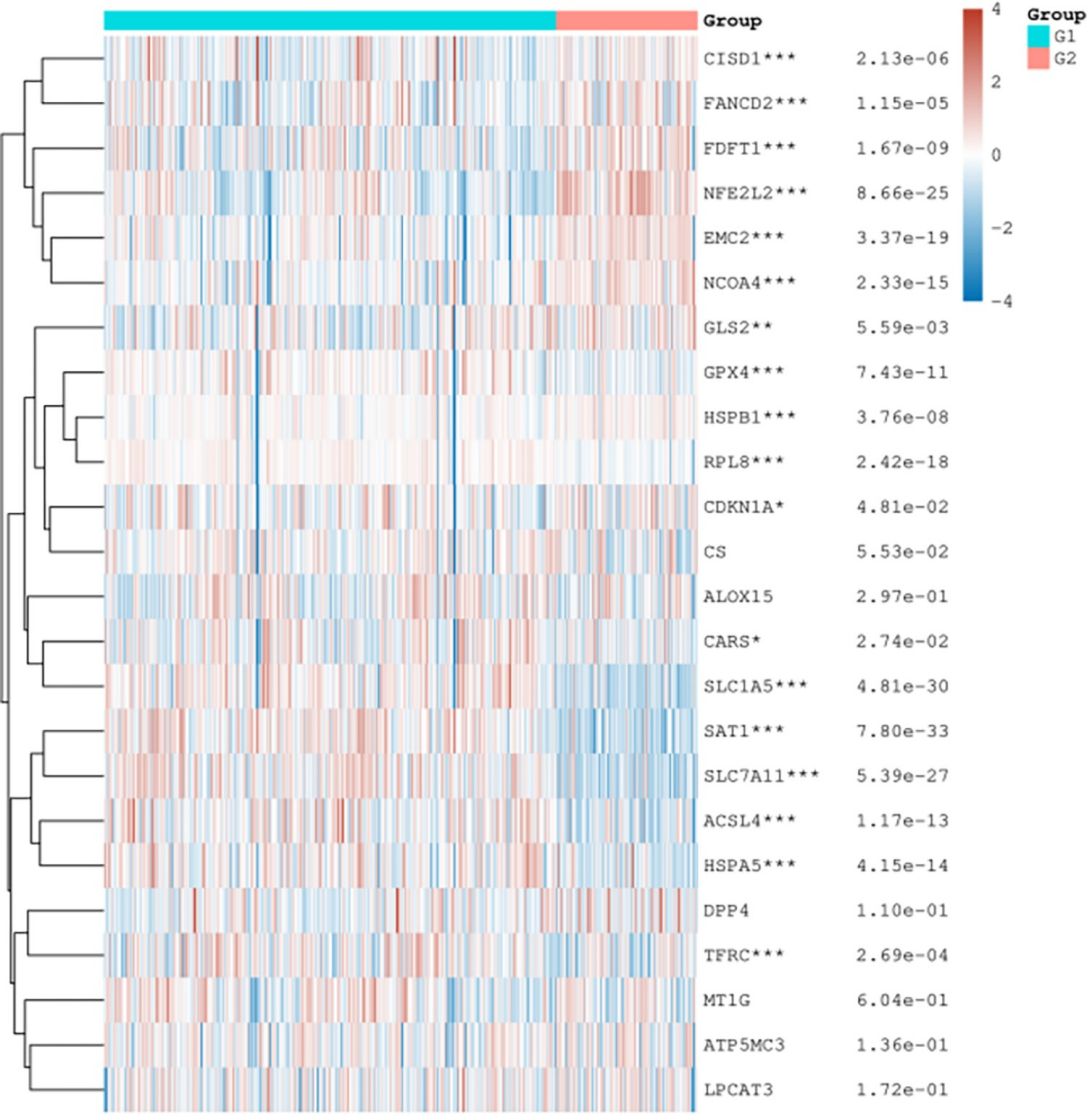
Ferroptosis-related gene expression heat map. The expression trend in different samples were represented by different colors. * p-value < 0.05, **p-value < 0.01, ***p-value < 0.001. The significance of the two groups passed the Wilcox test.

128 common DEGs in GSE16134 and GSE10334 between affected and unaffected groups were identified using R. We found that 101 DEGs were significantly upregulated and 27 DEGs were significantly downregulated (Table 3), the proportion was about 3.7:1. In this result, 8 ferroptosis-related genes related to periodontitis were up-regulated and 10 genes were down-regulated. The results after normalized data and individual horse effect removed are shown in Figs 1–3. And the frequency of *NCOA4*’s detection as the S1 Fig shows.

**Table 3:** Up/Downregulated DEGs.

Gene symbol	Adjusted P-value	Fold change	Gene title
*ADA2*	3.92E-57	1.553844	adenosine deaminase 2
*BHLHA15*	7.42E-58	1.588507	basic helix-loop-helix family, member a15
*BTG2*	1.07E-48	1.162589	BTG family, member 2
*C16ORF54*	3.83E-56	1.559668	chromosome 16 open reading frame 54
*C3*	9.22E-53	1.185639	complement component 3
*C4B*	1.95E-59	1.205283	complement component 4B (Chido blood group)
*CCL18*	1.34E-43	1.131173	chemokine (C-C motif) ligand 18
*CCN1*	3.80E-53	1.133135	cellular communication network factor 1
*CCN2*	4.85E-57	1.01127	cellular communication network factor 2
*CD177*	8.00E-57	1.096126	CD177 molecule
*CD19*	3.41E-64	1.192922	CD19 molecule
*CD27*	1.81E-45	1.813238	CD27 molecule
*CD38*	8.02E-43	1.551755	CD38 molecule
*CD79A*	5.19E-50	1.947502	CD79a molecule, immunoglobulin-associated alpha
*CFI*	6.18E-44	1.00665	complement factor I
*COL15A1*	3.12E-41	1.205063	collagen, type XV, alpha 1
*COL4A1*	4.04E-54	1.160181	collagen, type IV, alpha 1
*COL4A2*	3.09E-46	1.1936	collagen, type IV, alpha 2
*CORO1A*	8.85E-43	1.042673	coronin, actin binding protein, 1A
*CPNE5*	5.56E-53	1.308945	copine V
*CSF2RB*	1.49E-32	1.173406	colony stimulating factor 2 receptor, beta, low-affinity (granulocyte-macrophage)
*CSF3*	6.58E-29	1.259706	colony stimulating factor 3
*CTSH*	1.38E-52	1.034782	cathepsin H
*CXCL1*	9.72E-45	1.891672	chemokine (C-X-C motif) ligand 1 (melanoma growth stimulating activity, alpha)
*CXCL12*	1.96E-57	1.075039	chemokine (C-X-C motif) ligand 12
*CXCL13*	4.72E-53	1.392421	chemokine (C-X-C motif) ligand 13
*CXCL6*	2.39E-38	2.008496	chemokine (C-X-C motif) ligand 6
*CXCL8*	8.48E-35	1.275532	chemokine (C-X-C motif) ligand 8
*CXCR4*	4.07E-41	1.763415	chemokine (C-X-C motif) receptor 4
*CYP24A1*	1.45E-48	1.376211	cytochrome P450, family 24, subfamily A, polypeptide 1
*CYTIP*	6.14E-57	1.615435	cytohesin 1 interacting protein
*DDIT4L*	1.16E-60	1.051272	DNA-damage-inducible transcript 4-like
*DERL3*	5.72E-39	1.013822	derlin 3
*DNAJB9*	1.63E-53	1.022911	DnaJ (Hsp40) homolog, subfamily B, member 9
*DUSP5*	1.06E-36	1.049658	dual specificity phosphatase 5
*EGFL6*	2.65E-46	1.102904	EGF-like-domain, multiple 6
*ENPP2*	4.46E-49	1.133815	ectonucleotide pyrophosphatase/phosphodiesterase 2
*EVI2B*	3.21E-26	1.498001	ecotropic viral integration site 2B
*FCGR2B*	3.37E-37	1.249936	Fc fragment of IgG, low affinity IIb, receptor (CD32)
*FCGR3A*	1.95E-29	1.132553	Fc fragment of IgG, low affinity IIIa, receptor (CD16a)
*FCGR3B*	3.23E-56	1.342826	Fc fragment of IgG, low affinity IIIb, receptor (CD16b)
*FCN1*	3.76E-25	1.011778	ficolin (collagen/fibrinogen domain containing) 1
*FCRL5*	9.33E-45	1.455488	Fc receptor-like 5
*FCRLA*	1.09E-31	1.402519	Fc receptor-like A
*FKBP11*	1.04E-46	1.42036	FK506 binding protein 11
*FOS*	1.75E-44	1.290024	FBJ murine osteosarcoma viral oncogene homolog
*HBA1*	5.87E-36	1.14025	hemoglobin, alpha 1
*HCLS1*	2.79E-44	1.210277	hematopoietic cell-specific Lyn substrate 1
*HSPA13*	5.84E-58	1.159802	heat shock protein 70kDa family, member 13
*ICAM2*	1.63E-43	1.347935	intercellular adhesion molecule 2
*IGLL5*	3.07E-65	2.336454	immunoglobulin lambda-like polypeptide 5
*IL10RA*	3.18E-43	1.354325	interleukin 10 receptor, alpha
*IL1B*	2.82E-38	1.092466	interleukin 1 beta
*IL2RG*	6.34E-45	1.100459	interleukin 2 receptor, gamma
*ITM2C*	7.28E-47	1.184677	integral membrane protein 2C
*JCHAIN*	6.48E-49	1.286419	joining chain of multimeric IgA and IgM
*LAX1*	6.21E-59	1.652774	lymphocyte transmembrane adaptor 1
*LY96*	6.74E-45	1.12351	lymphocyte antigen 96
*MME*	9.12E-20	1.282694	membrane metallo-endopeptidase
*MMP1*	3.92E-57	1.025763	matrix metallopeptidase 1
*MMP12*	5.57E-59	1.466344	matrix metallopeptidase 12
*MMP13*	5.12E-54	1.178796	matrix metallopeptidase 13
*MMP3*	9.63E-57	1.040563	matrix metallopeptidase 3
*MMP7*	1.34E-34	1.51808	matrix metallopeptidase 7
*MZB1*	7.94E-43	2.407337	marginal zone B and B1 cell-specific protein
*NCF4*	1.49E-21	1.195206	neutrophil cytosolic factor 4
*ODAM*	1.33E-48	1.483623	odontogenic, ameloblast associated
*P2RY8*	1.21E-32	1.215359	purinergic receptor P2Y, G-protein coupled, 8
*PDZRN4*	2.52E-23	1.289339	PDZ domain containing ring finger 4
*PECAM1*	3.01E-28	1.098606	platelet/endothelial cell adhesion molecule 1
*PIM2*	4.14E-44	1.325032	Pim-2 proto-oncogene, serine/threonine kinase
*PLAC8*	3.25E-54	1.260461	placenta specific 8
*PLAT*	4.49E-46	1.43498	plasminogen activator, tissue
*PLPP5*	1.40E-51	1.0399	phospholipid phosphatase 5
*PNOC*	5.11E-71	1.387546	prepronociceptin
*POU2AF1*	6.04E-52	2.388694	POU class 2 associating factor 1
*PPBP*	1.82E-22	1.041058	pro-platelet basic protein
*PROK2*	4.58E-35	1.042763	prokineticin 2
*PTP4A3*	6.34E-57	1.07086	protein tyrosine phosphatase type IVA, member 3
*RAC2*	1.11E-43	1.173037	ras-related C3 botulinum toxin substrate 2 (rho family, small GTP binding protein Rac2)
*RGS1*	1.19E-45	1.196332	regulator of G-protein signaling 1
*RGS4*	3.12E-50	1.154445	regulator of G-protein signaling 4
*RHOH*	1.54E-47	1.262168	ras homolog family member H
*RNASE6*	8.98E-38	1.065063	ribonuclease, RNase A family, k6
*SASH3*	2.65E-37	1.005489	SAM and SH3 domain containing 3
*SEL1L3*	1.43E-54	1.097714	sel-1 suppressor of lin-12-like 3 (C. elegans)
*SELE*	4.59E-23	1.235928	selectin E
*SELENOM*	8.56E-43	1.199581	selenoprotein M
*SELL*	6.58E-22	1.458	selectin L
*SERPINI1*	6.35E-12	1.038916	serpin peptidase inhibitor, clade I (neuroserpin), member 1
*SFRP4*	9.20E-39	1.32865	secreted frizzled-related protein 4
*SLAMF7*	9.87E-41	1.619179	SLAM family member 7
*SPAG4*	5.26E-38	2.09838	sperm associated antigen 4
*SRGN*	1.36E-66	1.043883	serglycin
*ST6GAL1*	8.80E-11	1.051981	ST6 beta-galactosamide alpha-2,6-sialyltranferase 1
*TAGAP*	1.73E-34	1.11477	T-cell activation RhoGTPase activating protein
*TENT5C*	3.19E-45	2.014512	terminal nucleotidyltransferase 5C
*THEMIS2*	1.45E-45	1.330465	thymocyte selection associated family member 2
*TNFRSF17*	2.93E-31	2.349733	tumor necrosis factor receptor superfamily, member 17
*VCAN*	2.92E-39	1.038621	versican
*ZNF275*	2.71E-48	1.062687	zinc finger protein 275
*AADAC*	3.00E-42	-1.20712	arylacetamide deacetylase
*AADACL2*	1.08E-41	-2.01688	arylacetamide deacetylase-like 2
*ABCA12*	6.64E-33	-1.16284	ATP binding cassette subfamily A member 12
*ATP6V1C2*	1.23E-53	-1.14311	ATPase, H+ transporting, lysosomal 42kDa, V1 subunit C2
*BPIFC*	3.48E-44	-1.25704	BPI fold containing family C
*CALML5*	6.56E-27	-1.26108	calmodulin-like 5
*CLDN20*	6.70E-54	-1.28623	claudin 20
*COBL*	6.96E-56	-1.00726	cordon-bleu WH2 repeat protein
*DSC1*	1.76E-37	-2.14211	desmocollin 1
*ELOVL4*	1.09E-40	-1.25365	ELOVL fatty acid elongase 4
*EPCAM*	3.33E-36	-1.09877	epithelial cell adhesion molecule
*FAM83C*	3.12E-50	-1.02485	family with sequence similarity 83, member C
*FLG*	1.06E-18	-1.22646	filaggrin
*FLG2*	2.61E-27	-1.68284	filaggrin family member 2
*KRT1*	7.92E-11	-1.09036	keratin 1, type II
*KRT2*	3.04E-17	-1.5992	keratin 2, type II
*LCE2B*	1.67E-24	-1.0645	late cornified envelope 2B
*LOR*	1.67E-14	-1.32218	loricrin
*MAMDC2*	7.12E-24	-1.13528	MAM domain containing 2
*NEFL*	4.06E-49	-1.21988	neurofilament, light polypeptide
*NPR3*	3.87E-52	-1.09761	natriuretic peptide receptor 3
*POF1B*	1.36E-48	-1.08209	premature ovarian failure, 1B
*RPTN*	4.38E-15	-1.07675	repetin
*SLC16A9*	5.53E-43	-1.0713	solute carrier family 16, member 9
*SLC19A2*	8.89E-41	-1.04423	solute carrier family 19 (thiamine transporter), member 2
*SLC27A6*	2.00E-37	-1.26819	solute carrier family 27 (fatty acid transporter), member 6
*SPAG17*	4.10E-51	-1.19574	sperm associated antigen 17

### GO enrichment

**Fig 4:**
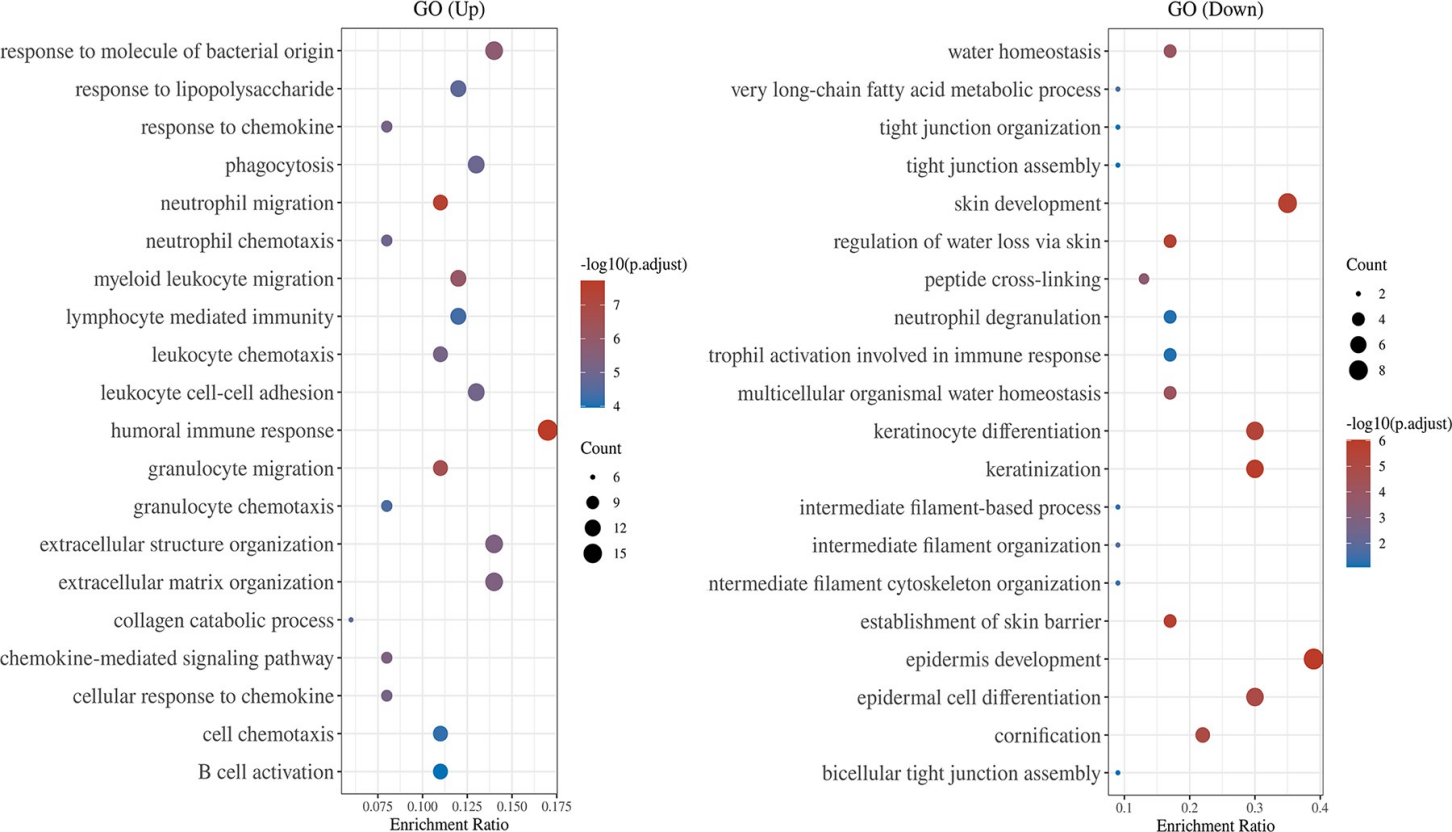
The abscissa represents gene ratio and the ordinate represents the enriched pathways. GO analysis of potential targets of mRNAs. The cellular component, biological process, and molecular function of FRGs were clustered based on Cluster Profiler package (R version: 3.18.0). In the enrichment result, p-value <0.05 or false discovery rate (FDR) <0.05 is considered to be enriched to a meaningful pathway.

GO (Fig 4) showed that upregulated DEGs were enriched for humoral immune response, response to molecule of bacterial origin, extracellular structure organization, phagocytosis, and leukocyte cell-cell adhesion. Downregulated DEGs were enriched for the epidermis development, skin development, keratinocyte differentiation, keratinization, and epidermal cell differentiation. The results of functional enrichment analysis of FRGs in normal tissues are shown in S1 Table.

### KEGG pathway enrichment analysis

**Fig 5:**
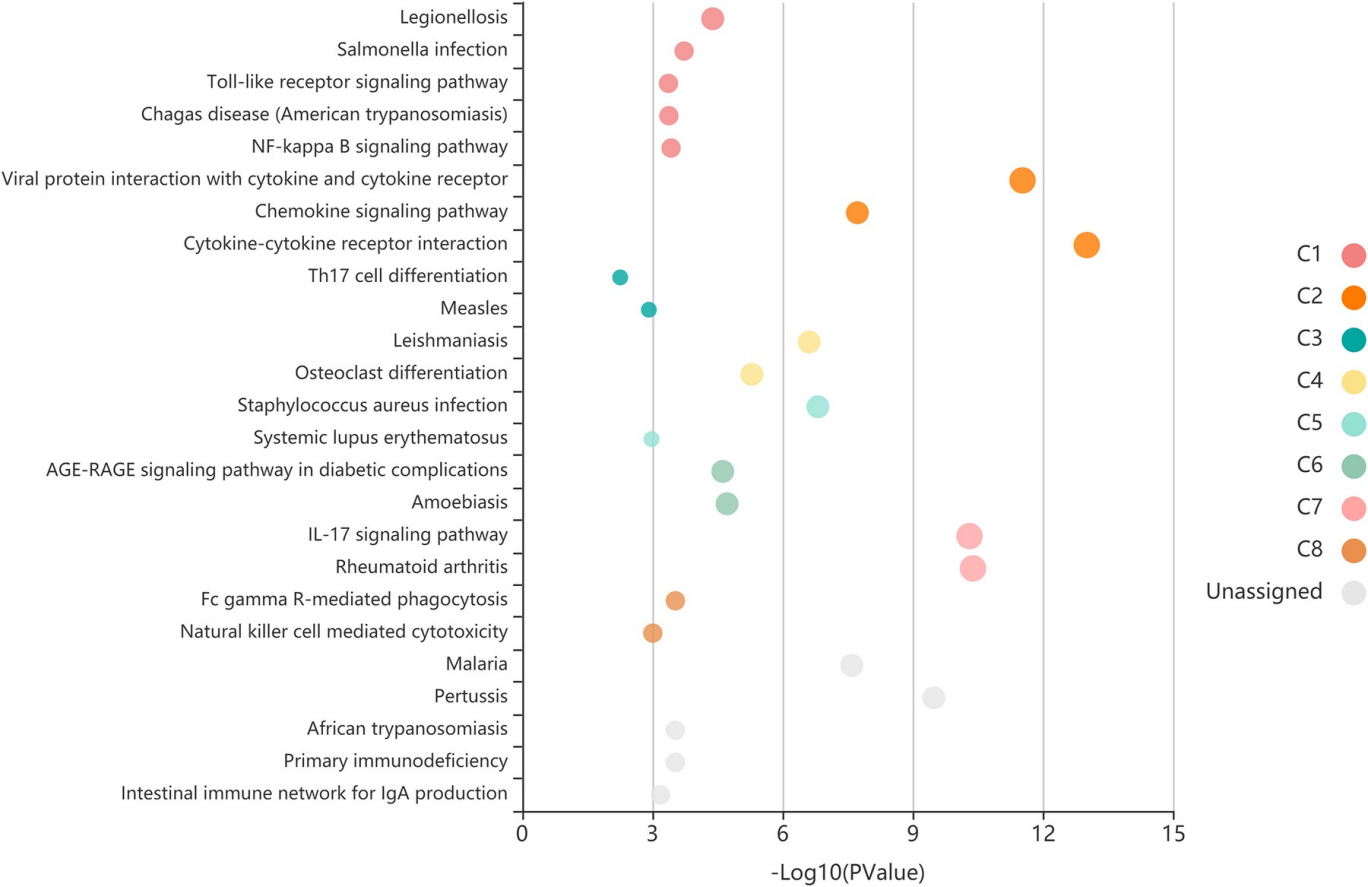
Each bubble which represents an enriched function is according to the number of input genes in this term. The size of the bubble represents the different significance levels. The different color of the bar and bubbles represent different modules, named C1-C8. If there are more than 5 terms in each module, top 5 with the highest enrich ratio will be displayed.

Fig 5 shows the result of KEGG pathway enrichment analysis. The five most regulated DEGs were those for the Cytokine-cytokine receptor interaction, Viral protein interaction with cytokine and cytokine receptor, Rheumatoid arthritis, IL-17 signaling pathway and Chemokine signaling pathway.

### GO-Biological process of ferroptosis-related genes

**Fig 6:**
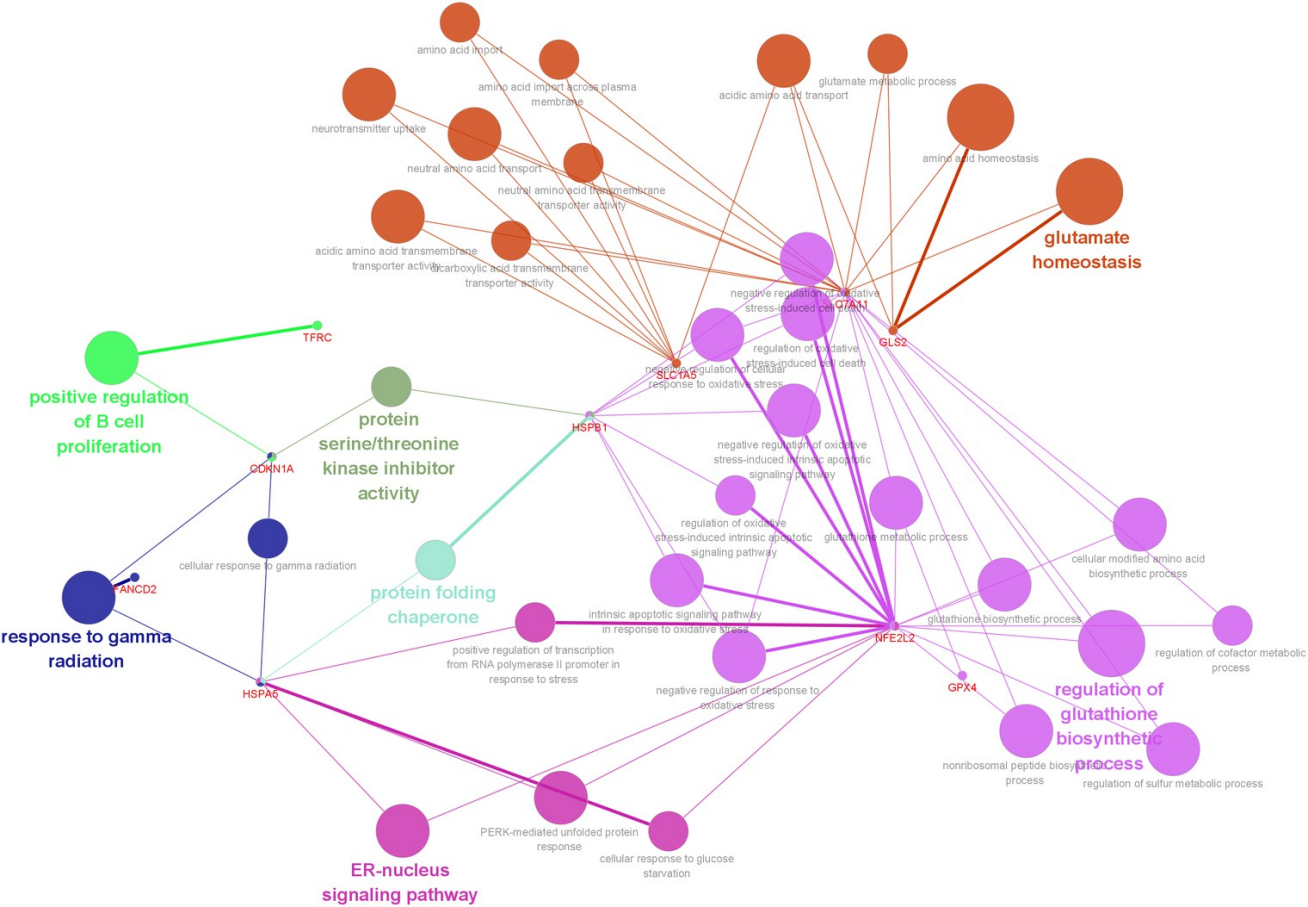
Pathways by using ClueGO analysis, p-value<0.05. Different pathways are represented by different colors.

It was found that overlapping targets of ferroptosis-related genes in periodontitis were involved in 34 pathways by using ClueGO analysis, including regulation of glutathione biosynthetic, glutamate homeostasis, ER-nucleus signaling pathway, response to gamma radiation, protein folding chaperone, positive regulation of B cell proliferation, protein serine/threonine kinase inhibitor activity, and so on (Fig 6).

### TF target analysis

**Fig 7:**
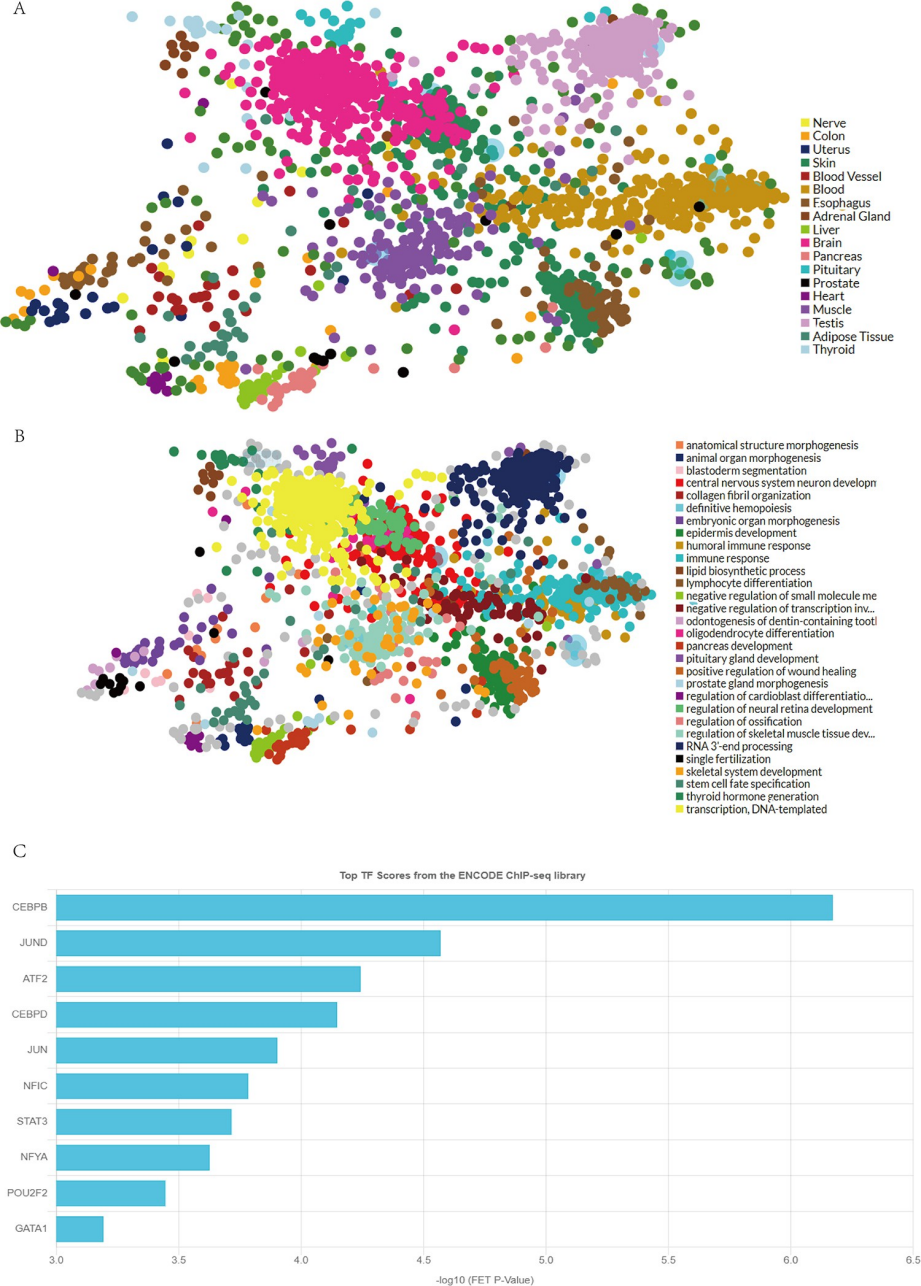
A: Various tissues, different tissues are represented by different colors; B: The functions of the TF targets, different functions are represented by different colors; C: The top 10 TFs.

The TFs were found to be distributed in various tissues, such as the muscle, brain, testis, and blood (Fig 7A). And the TF targets’ functions included immune response, DNA transcription, and skeletal muscle tissue development (Fig 7B). The top 10 TFs included *CEBPB*, *JUND*, *ATF2*, *CEBPD*, and *JUN* (Fig 7C).

### PPI analysis

**Fig 8:**
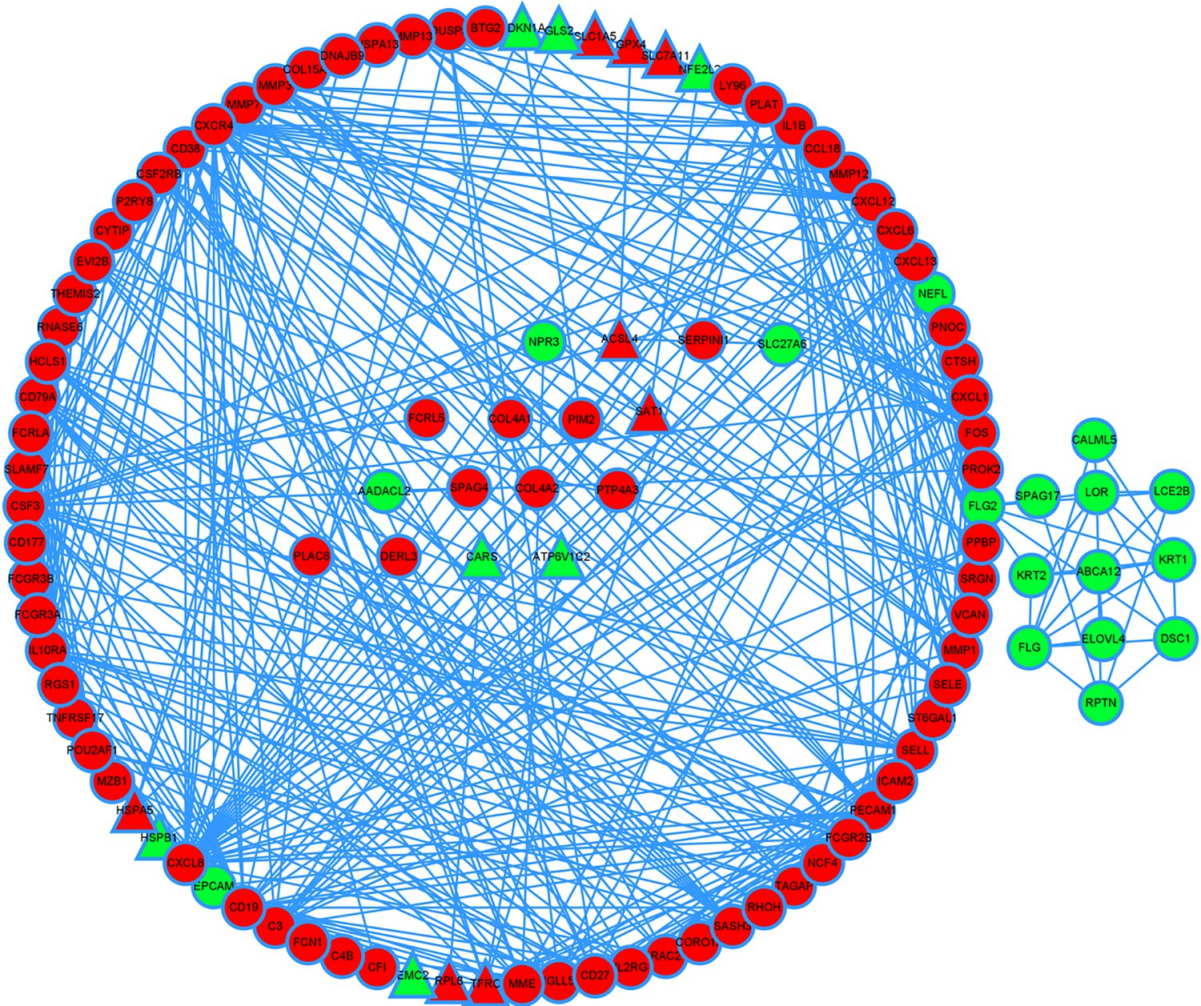
Significantly upregulated genes are marked in red and downregulated genes are marked in green. The hub nodes connected to FRGs are represented by triangles.

We used the Cytoscape and STRING databases to determin the greatest degree of network connection. The top ten hub genes identified were *CXCL8*, *CXCR4*, *IL1B*, *CD19*, *SELL*, *CXCL1*, *CXCL12*, *CSF3*, *PECAM1*, *FCGR2B*. And theTOP3 hub nodes connected to FRGs were *CXCL8*, *FOS*, *ATP5G3*. These genes were mainly linked to the negative regulation of IRE1-mediated unfolded protein response, regulation of type IIa hypersensitivity, regulation of apoptotic cell clearance, CXCR chemokine receptor binding, IgG binding, structural constituent of epidermis (Fig 8).

### ROC analysis

**Fig 9:**
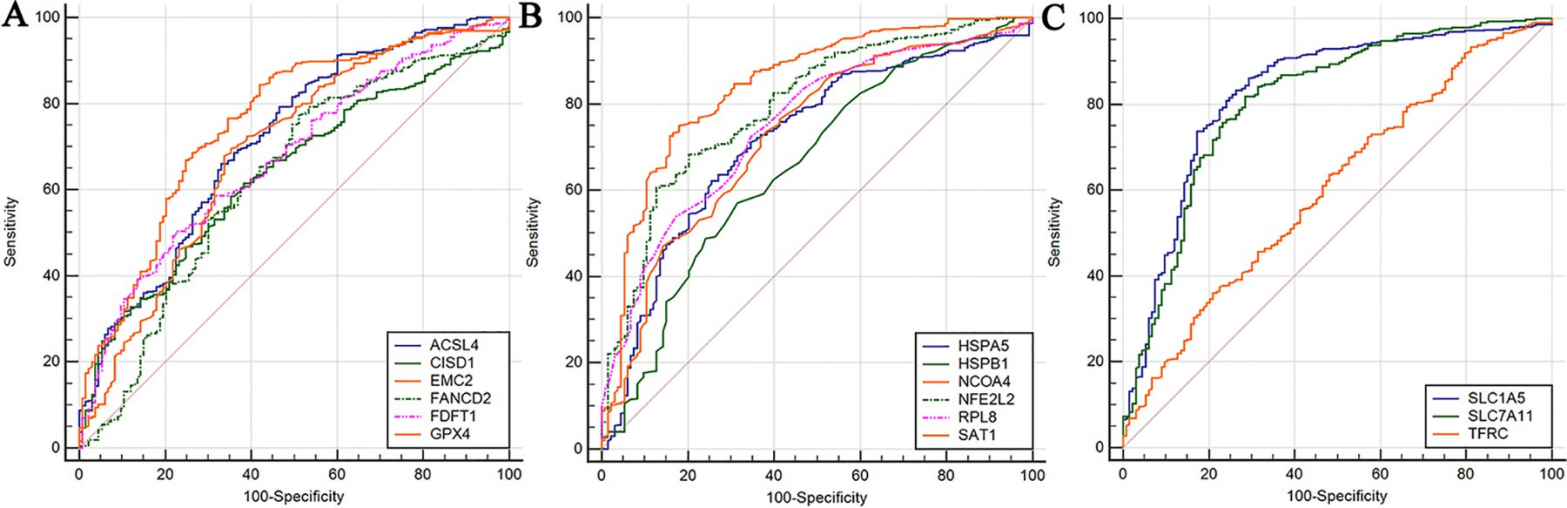
A: The ROC curves of *ACSL4*, *CISD1*, *EMC2*, *FANCD2*, *FDFT1* and *GPX4*. B: The ROC curves of *HSPA5*, *HSPB1*, *NCOA4*, *NFE2L2*, *RPL8* and *SAT1*. C: The ROC curves of *SLC1A5*, *SLC7A11* and *TFRC*.

Corresponding AUCs for FRGs were obtained, the diagnostic efficacy detected as “medium” level including *ACSL4* (AUC = 0.713), *EMC2* (AUC = 0.757), *HSPA5* (AUC = 0.717), *NCOA4* (AUC = 0.727), *NFE2L2* (AUC = 0.795), *RPL8* (AUC = 0.751), *SAT1* (AUC = 0.843), *SLC1A5* (AUC = 0.827), and *SLC7A11* (AUC = 0.809) (Fig 9).

### qRT-PCR experiment

**Fig 10:**
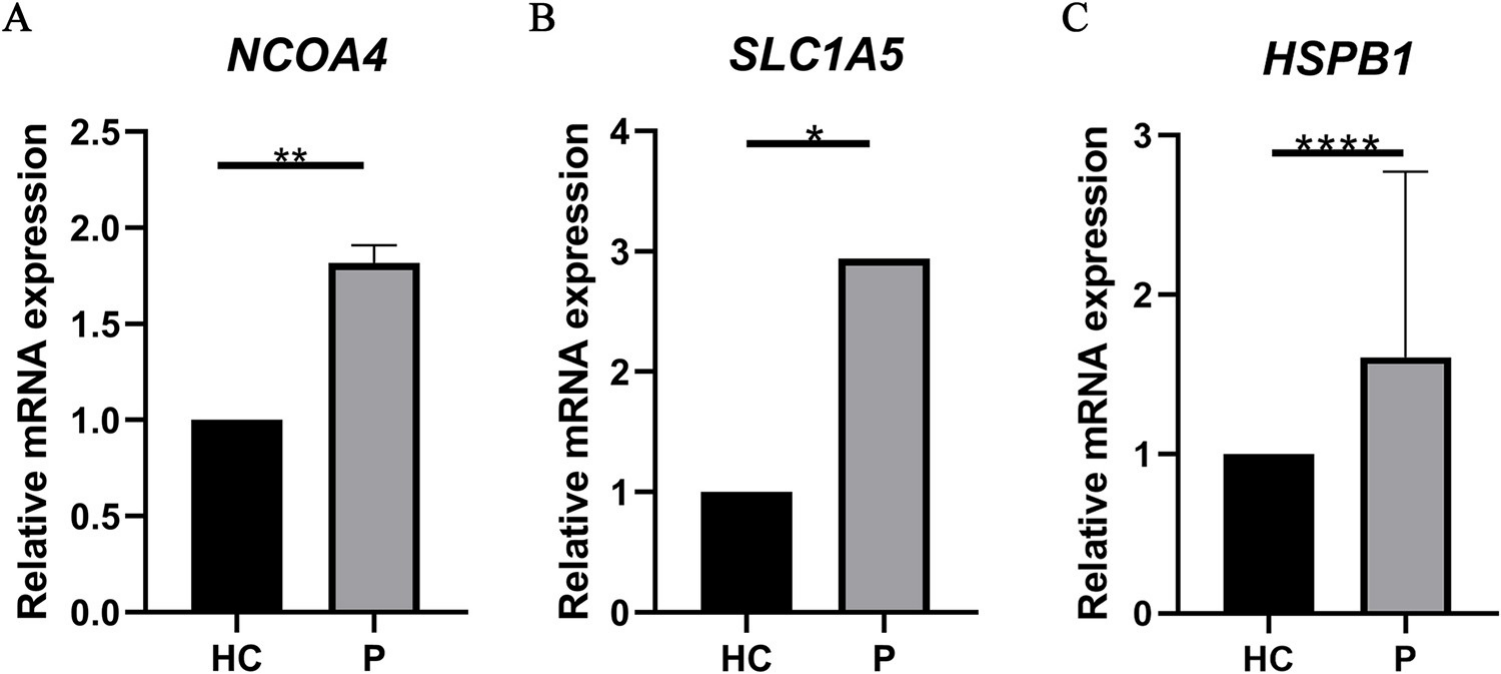
The expression levels of the three FRGs between healthy control group (n  =  6) and periodontitis group (n  =  6). All of the data are presented as means ± SD (p< 0.05).

To further confirm the bioinformatics analysis results, 6 normal gingival samples and 6 diseased samples were collected. Fig 10 shows the expression levels of *NCOA4*, *SLC1A5 and HSPB1* were significantly changed between normal gingival samples and diseased samples (p < 0.05).

## Discussion

Currently, various factors, including smoking, alcohol, metabolic syndrome, and obesity, were deemed to be the contributing factors of periodontitis [23]. However, understanding of the pathogenesis of the disease is still limited. Based on the RNA-seq, Davanian H confirmed that the elevated levels of locally produced cytokines in the periodontium [24]. A recent study including molecular biology experiments showed that NCOA4, one of the FRGs, is related to the occurrence and development of periodontitis, indicating that iron homeostasis plays an important role in the pathogenesis of periodontitis [20]. Ferroptosis triggers excess intracellular ROS and promotes lipid peroxidation, which is critical for inflammation. This study aimed to identify FRGs associated with periodontitis using bioinformatics analysis and may provide a deeper understanding of the link between ferroptosis and periodontitis. Also, this study has a potential value for the diagnosis, prognosis, and treatment of periodontitis. We analyzed two mRNA microarray datasets, obtained differentially expressed genes (DEGs), and screened FRGs from affected and unaffected group. A total of 128 DEGs and 18 screened FRGs were identified between the two datasets. Then, the DEGs and screened FRGs were subjected to GO and KEGG pathway analysis. PPI analysis of DEG and FRG was performed to enhance our understanding of the molecular mechanisms between periodontitis and ferroptosis, revealing a strong association between FRG and DEG at the protein level. To confirm the validity of these results, we conducted polymerase chain reaction (PCR) analysis for molecular biological verification at the gene level and ROC analysis to further understand the relationship between FRG and DEG.

We analyzed the pathway and functional enrichment of screened FRGs between the two groups as well as the results including glutathione biosynthetic. Some studies confirm that glutathione levels in patients with periodontitis are higher than those in healthy individuals, which is related to oxidative stress [25]. ROS regulates NF-κB responses, and one of the major signaling pathways that intersect with NF-κB in terms of ROS and cell death is the crosstalk that occurs between NF-κB and JNK [26]. According to various studies, glutathioneization regulates the activity of many transcription factors (TFs) through the binding of glutathione to the DNA-binding domain, which contains the TF NF-κB. Moreover, NF-κB plays an important role in the activation of pro-inflammatory responses [27,28].

The GO analysis results revealed that the upregulated DEGs were enriched in the humoral immune response, response to molecule of bacterial origin, and extracellular structure organization, whereas the downregulated DEGs were enriched in epidermis development, skin development, and keratinocyte differentiation. More and more evidence shows that cells dying by ferroptosis secrete factors that activate the immune system as expressed in the vivo pathological and genetic models of ferroptosis [29]. This can cause a periodontal immune response and reduce gingival development, clarifying the clinical manifestations of periodontitis. The KEGG pathway analysis indicated that the regulated DEGs were mainly enriched in chemokine signaling pathway [30,31]. In addition, we established a DEG and screened FRG PPI network, the hub genes include chemokines such as *CXCL8*, *CXCR4*, *CXCL1 and CXCL12*. And theTOP3 hub nodes connected to screened FRG were *CXCL8*, *FOS*, *ATP5G3*. Some studies have shown that chemokines are small proteins that secreted by cells that influence the immune system. *CXCL8* (interleukin-8) is a cytokine in the chemokine family [32] and an activator of neutrophils in inflammatory areas, which is released by gingival fibroblasts [33]. The final regulation of *CXCL8* gene expression depends on the ratio of CEBPB to NF-κB [34]. *CEBPB* is an important TF regulating the expression of immune and inflammatory response-related genes. Interestingly, *CEBPB* is one of the TF targets of FRGs in our study. We believe that *CEBPB* also plays an important role in periodontitis-related immune responses [35–37]. Meanwhile, researchers have found that *ATF2* is related to the occurrence of aggressive periodontitis [29]. These results suggest that TF targets and their signaling pathway genes are drivers of periodontitis progression. We also found that the analysis results of the downregulated genes include NCOA4, which is important in periodontitis. An article pointed out that periodontitis is mostly correlated with *NCOA4*-mediated ferritinophagy in intracellular iron level regulation [20], p38/hypoxia inducible factor-1α pathway activation, and bromodomain-containing protein 4 and cyclin-dependent kinase 9 transcription modulation mediated butyrate-triggered *NCOA4* expression in periodontal ligament fibroblasts [19]. Conversely, *NCOA4* over-expression increased sensitivity to ferroptosis [38]. It is fascinating that our biological experiments have proved that *NCOA4* is increased, and it also reminds us that transcriptomic datasets have certain drawbacks [39]. The difference in results is also because periodontitis is a disease affected by multiple factors [40]. We still need to further study the relationship between ferroptosis and periodontitis in the future. Through the comprehensive analysis of bioinformatic results, we speculated that ferroptosis promoted the occurrence and development of periodontitis through oxidative stress ROS and NF-κB related pathways.

The bioinformatic analysis of this study was based on published data and quantitative reverse-transcription PCR analysis with a relatively small number of samples to explore the relationship between FRGs and periodontitis. Further studies on the prediction and validation of periodontitis treatment targets including more samples and more forms of molecular biology validation are required.

## Conclusion

In summary, the present study determined the effect of ferroptosis in periodontitis. Ferroptosis provides new insights for understanding and preventing periodontitis. Better understanding of ferroptosis in periodontitis may aid in the diagnosis and treatment of periodontitis. Due to the limitations of bioinformatics itself, the findings of this study still need future experiments for further verification and research.

## Supporting information

S1 Fig (TIF)

S1 Table The role of FRGs. (XLSX)

S1 File The inclusion and exclusion criteria. (DOCX)

S1 Raw image GSE10334. (BMP)
