# Supplementary figures and images for: Role of ferroptosis-related genes in periodontitis based on integrated bioinformatics analysis
Source: PLoS One. 2022 Jul 28;17(7):e0271202. doi: 10.1371/journal.pone.0271202 (PMC9333299; doi:10.1371/journal.pone.0271202)

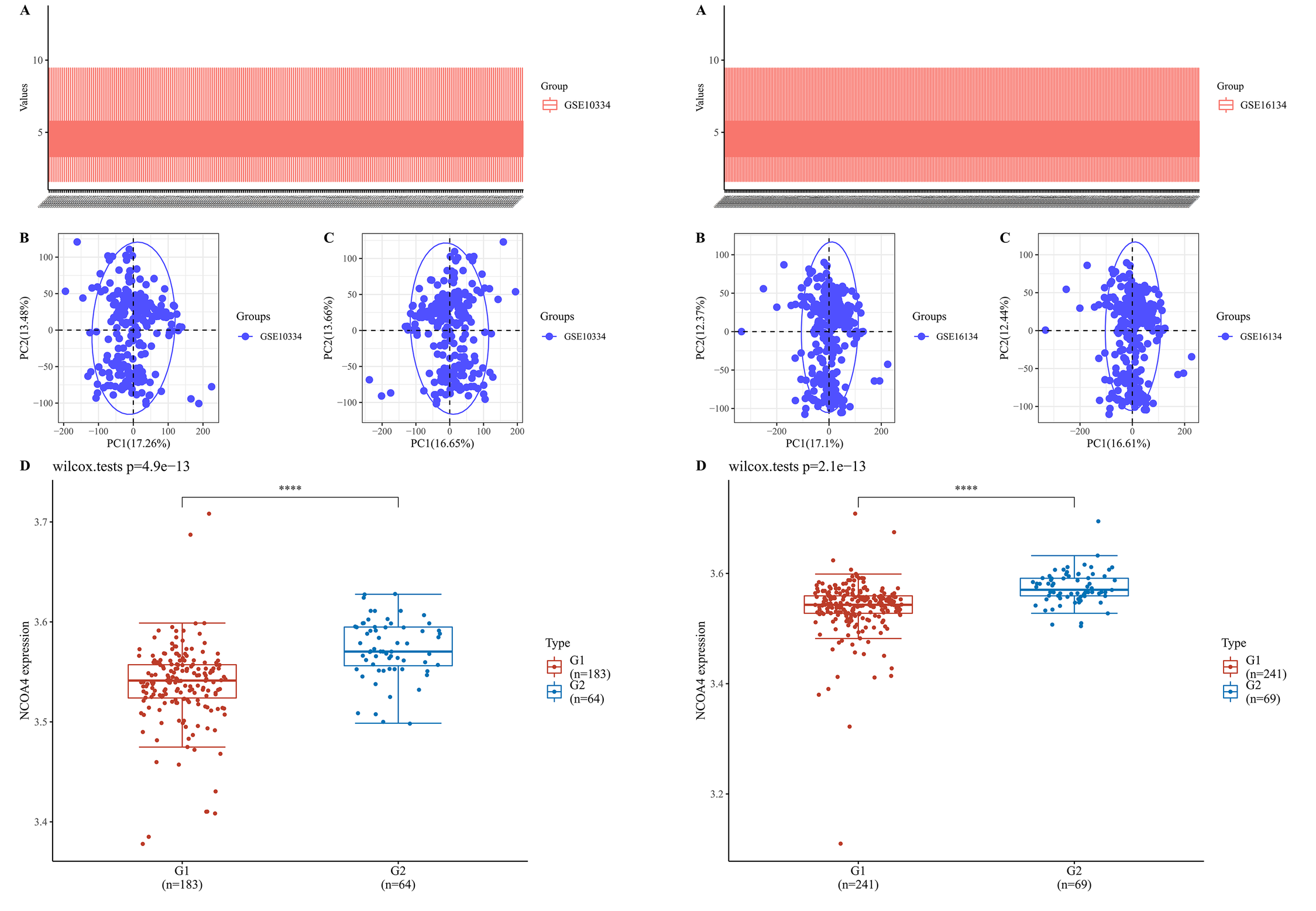

Supplement: S1 Fig — (TIF) [file pone.0271202.s001.tif]

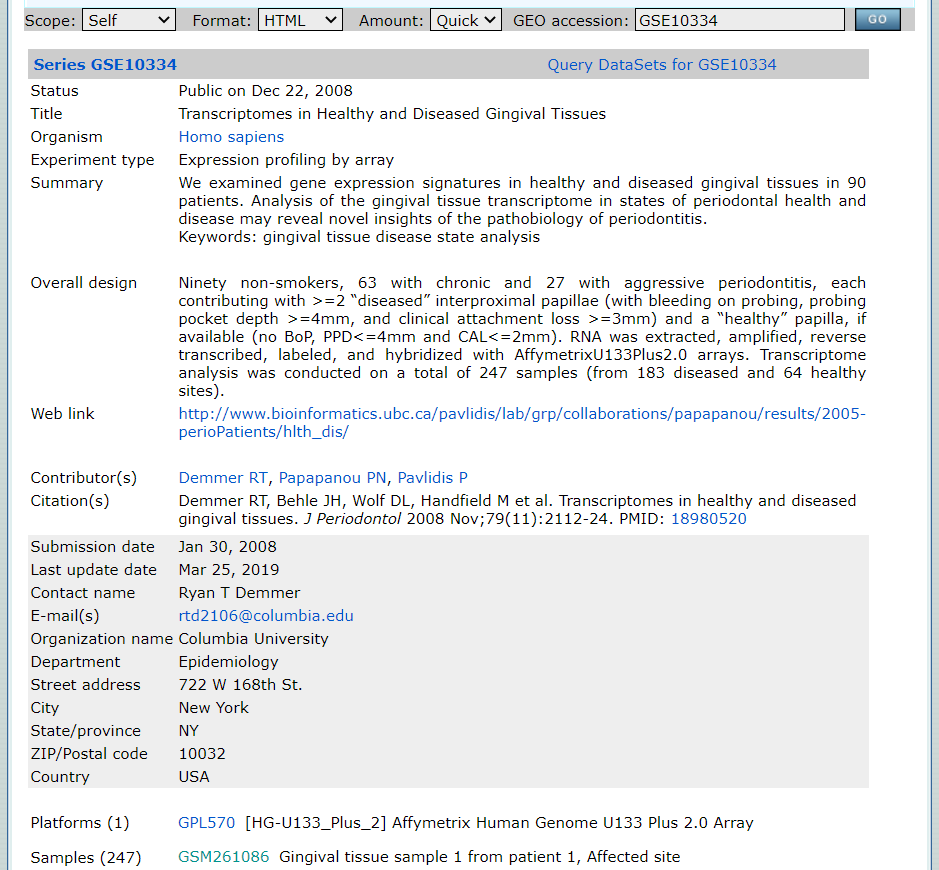

Supplement: S1 Raw image — (BMP) [file pone.0271202.s004.bmp]
