# Supplementary material for: Role of ferroptosis-related genes in periodontitis based on integrated bioinformatics analysis
Source: PLoS One. 2022 Jul 28;17(7):e0271202. doi: 10.1371/journal.pone.0271202 (PMC9333299; doi:10.1371/journal.pone.0271202)
Supplement: S1 File — (DOCX) [file pone.0271202.s003.docx]

Eligible patients were (i) at least 13 yr old; (ii) had a minimum of 24 teeth present; (iii) had no past history of systematic periodontal therapy other than occasional prophylaxis provided by the referring general dentist, (iv) had received no systemic antibiotics or anti-inflammatory drugs for at least 6 months, (v) harbored a minimum of 4 teeth with radiographic bone loss, (vi) did not suffer from diabetes mellitus, (vii) did not suffer from any of the systemic conditions or genetic disorders that entail a diagnosis of 'Periodontitis as a manifestation of systemic diseases', (viii) were not pregnant, and (ix) were not current users of tobacco products or of nicotine replacement medication. Signed informed consent was obtained prior to enrollment.

The PCR samples were collected from the interdental papilla of patients with periodontitis, the control group was taken from the hyperplastic interdental papilla of patients with dental implants. The samples obtained with a scalpel were immediately stored in liquid-nitrogen.
